# No-Reference Quality Assessment of In-Capture Distorted Videos

**DOI:** 10.3390/jimaging6080074

**Published:** 2020-07-30

**Authors:** Mirko Agarla, Luigi Celona, Raimondo Schettini

**Affiliations:** Department of Informatics, Systems and Communication, University of Milano-Bicocca, viale Sarca, 336, 20126 Milano, Italy; m.agarla@campus.unimib.it (M.A.); raimondo.schettini@unimib.it (R.S.)

**Keywords:** video quality assessment, in-capture distortions, convolutional neural network, recurrent neural network

## Abstract

We introduce a no-reference method for the assessment of the quality of videos affected by in-capture distortions due to camera hardware and processing software. The proposed method encodes both quality attributes and semantic content of each video frame by using two Convolutional Neural Networks (CNNs) and then estimates the quality score of the whole video by using a Recurrent Neural Network (RNN), which models the temporal information. The extensive experiments conducted on four benchmark databases (CVD2014, KoNViD-1k, LIVE-Qualcomm, and LIVE-VQC) containing in-capture distortions demonstrate the effectiveness of the proposed method and its ability to generalize in cross-database setup.

## 1. Introduction

In recent years, the increase in devices such as smartphones and tablets has led to an exponential growth in the amount of videos captured and shared on social media like YouTube, Snapchat, Facebook, and Instagram [1,2]. Different forms of distortions can be introduced during the video acquisition and transmission processes. The distortions introduced by the camera hardware and processing software during the capture process are called in-capture, while the distortions like compression and transmission errors are called post-capture distortions. The automatic estimation of the quality of a digital video as perceived by human observers can be relevant for a wide range of applications. For example, to discriminate professional and amateur video content on user-generated video distribution platforms, to guide a video enhancement process, and to rank/choose user-generated videos.

Video Quality Assessment (VQA) aims at the development of methods that produce quality predictions in close agreement with human judgments, regardless of the video contents and the type and the severity of the distortions that have corrupted the videos. VQA methods can be classified into three main categories: Full-Reference (FR) [3,4,5,6], Reduced-Reference (RR) [7,8] and No-Reference (NR) [9,10,11,12,13,14] depending on whether all or part of the pristine reference video is used in the assessment process. Existing NR-VQA methods can be further grouped based on whether they model only frame-level features or also explicitly account for frame temporal information. Frame-based NR-VQA methods are mostly based on image quality assessment methods and involve the analysis of Natural Scene Statistics (NSS). Among such methods there are the Naturalness Image Quality Evaluator (NIQE) [9], the Blind/Referenceless Image Spatial Quality Evaluator (BRISQUE) [13], the Feature maps based Referenceless Image QUality Evaluation Engine (FRIQUEE) [14], and the High Dynamic Range Image Gradient based Evaluator (HIGRADE) [15]. All NSS-based methods applied to videos independently measure the deviation of frames from the natural scene statistics and then aggregate these statistics by calculating the average to obtain the quality score for the entire video. Few methods in the literature explicitly consider temporal features. V-BLIINDS [12] extends the image-based metric by incorporating time-frequency characteristics and temporal motion information. The Video Codebook Representation for No-Reference Image Assessment (V-CORNIA) [14] is an unsupervised frame-feature learning approach that uses Support Vector Regression (SVR) to predict frame-level quality, then the final video quality is obtained by temporal pooling.

Recent algorithms for NR-VQA involve the use of Deep Neural Networks (DNNs). SACONVA [16] is based on a 3D shearlet transform for extracting frame-level features, which are then passed to a 1D Convolutional Neural Network (CNN) to predict spatio-temporal quality features. The COnvolutional neural network and Multi-regression based Evaluation (COME) [17] splits the problem of extracting spatio-temporal quality features into two parts. At first, spatial quality features are extracted for each video frame by both max pooling and computing standard deviation of the final activation layer of an AlexNet pre-trained for image quality assessment on the CSIQ dataset [18]. Temporal quality features are then extracted as standard deviation of motion scores in the video. Finally, two SVR are used in conjunction with a Bayes classifier to predict the video quality score. VSFA [11] integrates into a DNN two eminent effects of the human visual system, namely content dependency and temporal memory effects. Specifically, VSFA extracts content-aware features from a CNN pre-trained on ImageNet [19] and then it includes a Gated Recurrent Unit (GRU) [20] for modeling long-term dependencies and predicting frame quality. Finally, to take the temporal hysteresis effects into account, it contains a differentiable subjectively-inspired temporal pooling model to output the overall video quality. VSFA demonstrated to be very effective on three benchmark video databases containing in-capture distortions, i.e., KoNViD-1k [21], CVD2014 [22], and LIVE-Qualcomm [23]. Recently, the Two Level Video Quality Model (TVLQM) [24] has been presented, it consists of a two level feature extraction mechanism in which low complexity features are first computed for the full sequence, and high complexity features are then extracted from a subset of representative video frames.

In this paper we focus on the problem of assessing the quality of videos affected by distortions introduced during the capture process. The proposed method relies on the rationale that human judgments of visual video quality depend on: the semantic content [25,26], the different sensitivity to low-level visual psychological characteristics [27], and the temporal-memory effects [11]. Image regions presenting clear semantic information are more sensitive to the presence of impairments, consequently they may be judged as more annoying by humans as they hinder the content recognition [25]. Encoding the semantic content of frames is then crucial to align better with human perception. The human visual system (HVS) is differently sensitive to some visual phenomena including luminance nonlinearity [28] and contrast sensitivity [29]. Thus, it is important to consider these aspects while measuring the overall quality. Temporal-memory effects indicate that human judgments of the current frame are based on the current frame and information from previous frames. This implies that long-term dependencies exist in the VQA problem. In more detail, humans more easily remember past poor quality frames and lower perceived quality scores for subsequent frames, even when frame quality has returned to acceptable levels. This effect is called temporal hysteresis and its modeling demonstrated to be effective in previous VQA methods [11,30]. To take into account the aforementioned phenomena, we propose the Quality and Semantics Aware Video Quality Method (QSA-VQM). It consists of two main blocks, namely the Multi-level feature extraction block and the Temporal modeling block. The Multi-level feature extraction block involves the use of two Convolutional Neural Networks (CNNs) for encoding a frame at a time in terms of both semantic and quality features. The Temporal modeling block is then in charge of estimating the overall quality score for the video by combining frame features thanks to a Recurrent Neural Network (RNN) [31,32] and a Temporal Hysteresis Pooling [11]. The proposed QSA-VQM is inspired by the VSFA [11], in which the use of the two main blocks is already proposed, namely the Multi-level feature extraction and the Temporal modeling. In the QSA-VQM as in the VSFA, the frames are encoded using deep features extracted from a network trained for object categorization. These deep features are content-aware and sensitive to the distortions at the same time [33]. We differentiate ourselves from the VSFA because, in addition to the previous features, we describe the video frames with a network trained to estimate different quality attributes, which provides a better representation of those peculiarities that are needed to characterize the quality of videos. We also improve the Temporal modeling part by replacing the GRU used in VSFA with the RNN, which has proven to correlate better with MOS.

The main contributions of this work are the following:

- A no-reference video quality assessment method for in-capture distortions using two CNNs for encoding video frames in terms of both semantics and quality attributes, and a temporal modeling block including a Recurrent Neural Network (RNN) and a Temporal Hysteresis Pooling layer.
- An evaluation of the proposed method with previous VQA methods on four benchmark databases containing in-capture distortions also in cross-database setup.
- An ablation study measuring the advantages of combining semantics and quality features and the impact of using alternative approaches to RNN for temporal modeling.

The rest of the paper is organized as follows. In Section 2, the proposed method is detailed. Section 3 describes the databases and the training protocol. Section 4 shows all the experimental results and an ablation study where all the investigated methods that allow the definition of the final method are compared. Finally, Section 5 concludes and discusses some possible future work.

## 2. The Quality and Semantics Aware Video Quality Method

**Figure 1:**
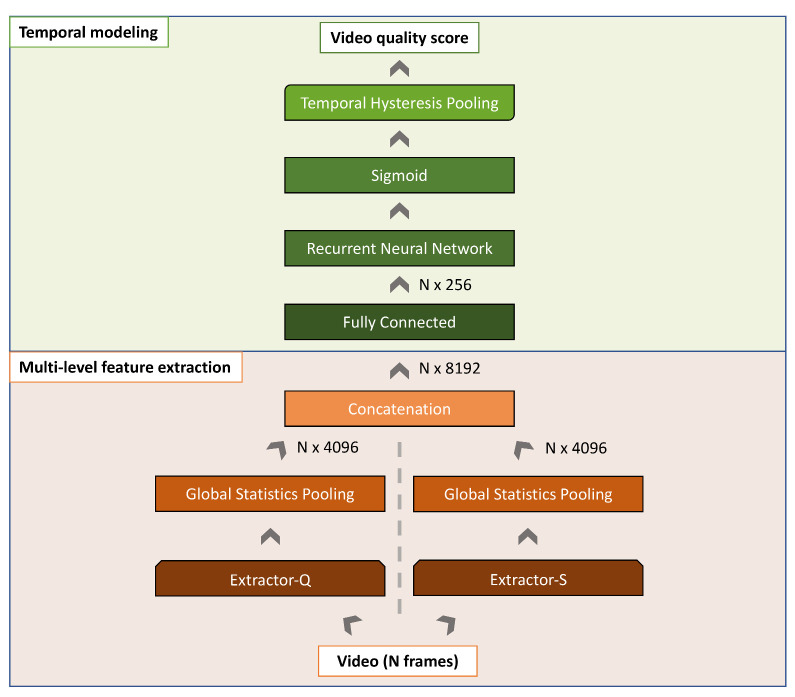
The proposed Quality and Semantics Aware Video Quality Method (QSA-VQM). The QSA-VQM consists of two main blocks: the Multi-level feature extraction and the Temporal modeling. The Multi-level feature extraction block encodes each video frame in terms of both quality and semantic features. The Temporal modeling block maps frame-level feature vectors into a frame-level quality score using a Recurrent Neural Network (RNN) and then combines all the quality scores thanks to a Temporal Hysteresis Pooling.

The proposed method we called Quality and Semantics Aware Video Quality Method (QSA-VQM) is depicted in Figure 1. It estimates the quality score of RGB video sequences of variable resolution and length. It consists of two main blocks: the Multi-level feature extraction block and the Temporal modeling block. In the Multi-level feature extraction block, video frames are fed one at a time into two Convolutional Neural Networks (CNNs), called Extractor-Q and Extractor-S, in which the aim is to compute quality and semantic features for each video frame. These features are concatenated and then processed by the Temporal modeling block, which involves a Fully Connected (FC) layer for dimensionality reduction, and a Recurrent Neural Network (RNN) layer [31,32], which predicts a quality score for each frame also taking into account previous frames. The quality score for the whole video is finally obtained by applying the Temporal Hysteresis Pooling proposed in [11] on the scores previously predicted for video frames. In the next sections we detail each block of the QSA-VQM.

### 2.1. Multi-Level Feature Extraction

Given that human judgments of visual video quality are strongly influenced by the different sensitivity to low-level visual psychological characteristics [27,34] and the semantic video content [25,26] in this work we characterize video frames in terms of these two aspects. To this end, we employ two CNNs that we have called Extractor-Q and Extractor-S to extract quality and semantic features, respectively.

**Figure 2:**
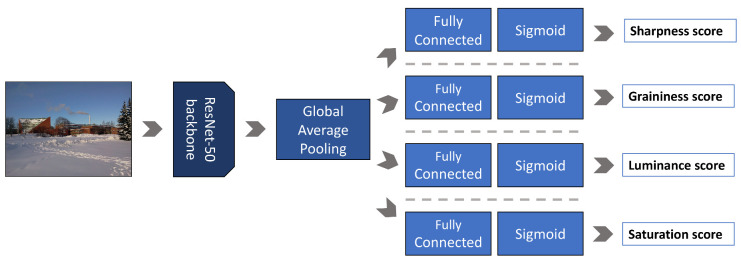
Definition of the Extractor-Q. Given an image of any size the Extractor-Q simultaneously estimates the scores for four quality attributes, namely sharpness, graininess, lightness, and color saturation. It consists of a ResNet-50 followed by a Global Average Pooling (GAP) and a stack of fully connected (FC) + sigmoid layers for each task.

In Figure 2 we show the architecture of the Extractor-Q. It consists of a ResNet-50 [35] architecture (given its good trade-off between performance and number of parameters [36]) truncated to the last convolutional layer, then a Global Average Pooling (GAP) layer that is followed by four different Fully Connected (FC) layers. The sigmoid activation function is finally applied to each output layer because we want predicted scores to lie in the range [0,1] according to MOS. The Extractor-Q is end-to-end trained in a multi-task fashion for simultaneously estimating sharpness, graininess, lightness, and color saturation of images. To train the Extractor-Q we use the CID2013 database [37], which consists of 480 images captured by 79 different cameras of varying quality. Each image is annotated by human subjects in terms of overall quality and four attribute scales (i.e., sharpness, graininess, lightness, and color saturation). During training, the input images are not resized before feeding into Extractor-Q so that the network is trained on images having the same resolution as that used for collecting annotations. The resize of the image could in fact introduce interpolation artifacts.

The semantic features for each frame are simply obtained using the Extractor-S, which consists of a ResNet-50 pre-trained on ImageNet for image categorization.

The feature vector for each of the *N* video frames from both CNNs is obtained by truncating the networks to the last convolutional block, which generates an activation volume of m×n×2048, where m×n is the spatial resolution and 2048 is the depth of the volume, respectively. A Global Statistics Pooling (GSP) [38] is then applied by calculating and concatenating the mean and standard deviation of spatial features. The feature vector obtained by a GSP has shape N×4096. The output of the Multi-level feature extraction block is obtained by concatenating the feature vectors of each network then getting a video frame representation of N×8192.

### 2.2. Temporal Modeling

We use a Recurrent Neural Network (RNN) layer [32] to mimic the viewer memory watching the video. The ability of RNN is to represent dependencies over the time using an internal hidden state, $h_{t}$, which is computed as follows:

(1) $$\begin{array}{cc} h_{t} & =\tanh(W_{ih}x_{t}+b_{ih}+W_{hh}h_{t-1}+b_{hh}). \end{array}$$

The current feature vector ($x_{t}$) is combined with the previous hidden state ($h_{t-1}$) thanks to the input-hidden weights ($W_{ih}$) and bias ($b_{ih}$), and the hidden-hidden weights ($W_{hh}$) and bias ($b_{hh}$). The output state $y_{t}$, which represents the predicted quality score for the current frame, is then obtained using a fully connected layer with parameters $W_{hy}$ and $b_{hy}$:

(2) $$\begin{array}{cc} y_{t} & =W_{hy}h_{t}+b_{hy}. \end{array}$$

**Figure 3:**
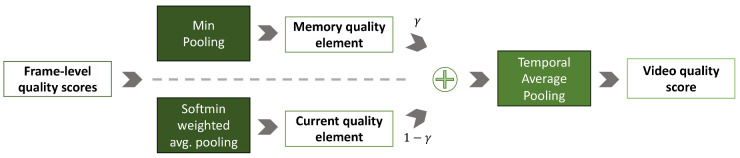
Temporal Hysteresis Pooling [11]. This pooling layer addresses the problem of overestimating the quality of videos. Specifically, a Memory quality element is defined as the minimum of the quality scores over the previous frames, while a Current quality element is defined as a sort-order-based weighted average of the quality scores over the next frames. The approximate score is then calculated as the weighted average (γ) of the Memory and Current elements. The overall video quality is finally computed as the temporal average pooling of the approximate scores.

Since a long feature vector could produce overfitting and high computation time in RNNs, a fully connected layer is applied for reducing frame-level representations from 8192 dimensions to 256. The RNN takes the frame-level representations as input for estimating the corresponding quality scores that are then processed by a sigmoid activation function to keep them into the range [0, 1] that is the same range of ground-truth MOS. Finally, the frame-level scores are aggregated to obtain the overall video quality score thanks to a Temporal Hysteresis Pooling [11], which limit the temporal hysteresis effect (see Figure 3). The temporal hysteresis effect is due to the fact that viewers react sharply to drops in video quality and react dully to any improvement. In the Min pooling block of the Temporal Hysteresis Pooling, the memory quality follows the viewer intolerance to poor quality events:

(3) $$\begin{array}{c} l_{t}=\left\{\begin{array}{cc} q_{t}, & \mathrm{if}\;t=1\\ \min_{\forall k\in V_{prev}}q_{k}, & \mathrm{otherwise}, \end{array}\right. \end{array}$$

where $l_{t}$ is the memory quality element at time *t*, $q_{t}$ is the frame quality at time *t*, $V_{prev}=max(1,t-\tau ),\ldots ,t-2,t-1$ are the quality scores of the previous τ frames, and τ is a hyper-parameter related to temporal duration. The other block of the Temporal Hysteresis Pooling, say the Softmin weighted average pooling, assigns larger weights to worse quality frames for obtaining the current quality element, $m_{t}$:

(4) $$\begin{array}{cc} m_{t} & =\sum_{k\in V_{next}}q_{k}w_{t}^{k} \end{array}$$

(5) $$\begin{array}{cc} w_{t}^{k} & =\frac{e^{-q_{k}}}{\sum_{j\in V_{next}}e^{-q_{j}}},\;k\in V_{next} \end{array}$$

where $w_{t}^{k}$ is the $t^{th}$ quality weight score represented by a differentiable Softmin function, $V_{next}=t,t+1,\ldots ,min(t+\tau ,T)$ are the quality scores of the next τ frames. The overall quality score for the video, *Q*, is obtained thanks to the linear combination of memory quality, $l_{t}$, and current quality elements, $m_{t}$:

(6) $$\begin{array}{cc} Q & =\frac{1}{T}\sum_{t=1}^{T}q_{t}^{\prime } \end{array}$$

(7) $$\begin{array}{cc} q_{t}^{\prime } & =\gamma l_{t}+\left(1-\gamma \right)m_{t}. \end{array}$$

In this case γ hyper-parameter balances the contributions of memory and current elements to the approximate score.

### 2.3. Implementation Details

We develop our method, which consists of several training processes using the PyTorch framework [39]. The first two trainings concern the two CNNs of the Multi-level feature extraction block, while the other training regards only the Temporal modeling block.

For the Extractor-S we use the ImageNet pre-trained ResNet-50 provided by the Torchvision package of the PyTorch framework [39]. As mentioned in Section 2.1, the Extractor-Q is trained on the CID2013 database for the estimation of quality attributes. Also in this case we start from a ResNet-50 pre-trained on Imagenet and we use the initialization technique proposed in [40] for the fully connected layers predicting the scores for each attribute. Image labels for each quality attribute are mapped in range [0,1] using the min–max scaling. Adam is chosen as the optimizer, while the linear combination of a Mean Absolute Error (MAE) loss for each task is used as optimization criterion. The learning rate is set to $1\times 10^{-6}$, and the weight decay is set to $1\times 10^{-5}$ to prevent overfitting. We train the network for 24 epochs on the entire dataset with batch size equal to 4.

The temporal modeling block gets the concatenated features from the two extractors, i.e., the Extractor-S and the Extractor-Q, as input and is trained for estimating the overall video quality score. The RNN has a single layer and a hidden state of 64 features. In the Temporal hysteresis block, we set τ to 12 and γ to 0.5 because along experiments presented in [11], these were demonstrated to be the best parameters. Batch size is set to 4, the optimizer is Adam and MAE is used as the loss function. Before MAE estimation, ground-truth MOS are scaled in the range [0, 1] using the min–max scaling. The learning rate is set to $1\times 10^{-5}$ and it decays of 0.8 every 50 epochs. Early stopping criterion is applied when validation Spearman’s Rank-Order Correlation Coefficient (SROCC) does not improve after 50 epochs on the validation set. Epochs limit is set to 300.

## 3. Experiments

In this section, we first describe the databases considered for the experiments, we then present the experimental setup and the evaluation criteria.

### 3.1. Database with In-Capture Video Distortions

Four publicly available databases are widely used for video quality assessment in-the-wild, namely: Camera Video Database (CVD2014) [22], Konstanz Natural Video Database (KoNViD-1k) [21], LIVE-Qualcomm Mobile In-Capture Video Quality Database (LIVE-Qualcomm) [23], and LIVE Video Quality Challenge Database (LIVE-VQC) [41].

The CVD2014 database [22] consists of 234 videos of resolution 640×480 or 1280×720 recorded by 78 different cameras (from low-quality mobile phone cameras to high-quality digital single lens reflex cameras). Each video captures one among five different scenes and presents distortions related to the video acquisition process. The length of the trimmed videos is 10–25 s with 11–31 fps. The realignment MOS scores lay in the range [−6.50, 93.38].

The KoNViD-1k database [21] is a collection of 1200 videos of resolution 960×540 sampled according to six specific attributes from the YFCC100M dataset [42]. The resulting database contains video sequences that are representative of a wide variety of contents and authentic distortions. The videos are 8 s with 24/25/30 fps. The MOS have been collected through a crowdsourcing experiment and range from 1.22 to 4.64.

The LIVE-Qualcomm database [23] includes 208 videos of resolution 1920×1080 captured by 8 different smartphones. These videos have a length of 15s and are affected by 6 in-capture distortions, i.e., artifacts, color, exposure, focus, sharpness, and stabilization. A subjective study was conducted under two different study protocols in a controlled laboratory. A total of 39 subjects were randomly assigned to one of the setups. The unbiased study setup aimed to gather quality scores while the subjects freely watch videos, while in the biased (distortion-guided) study the subjects were already informed of which type of distortion corrupted the video. In this work we consider the unbiased MOS scores, which belong to the range [16.56, 73.64].

Finally, the LIVE Video Quality Challenge (LIVE-VQC) database [41] contains 585 videos of unique content, captured by 101 different devices (the majority of these were smartphones), with a wide range of complex authentic distortions. Predominant resolutions are 404×720, 1024×720, and 1920×1080. Videos duration is 10 s on average. Subjective video quality scores were collected via crowdsourcing: a total of 4776 unique participants produced more than 205,000 opinion scores. MOS span between 0 and 100.

**Figure 4:**
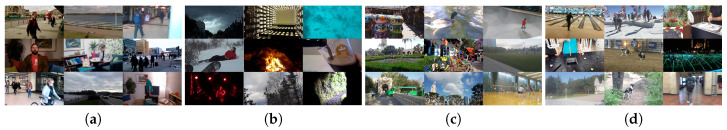
Sample frames of the video contents contained in the four in-capture databases: (**a**) CVD2014, (**b**) KoNViD-1k, (**c**) LIVE-Qualcomm, and (**d**) LIVE-VQC.

Frame samples are in Figure 4, while an overview of database properties is provided in Table 1.

### 3.2. Experimental Setup

The evaluation metrics for no-reference video quality assessment methods are the same as those commonly used for no-reference image quality assessment, namely: Pearson’s Linear Correlation Coefficient (PLCC), Spearman’s Rank-order Correlation Coefficient (SROCC), and Root Mean Square Error (RMSE).

The PLCC measures the linear correlation between the actual and the predicted scores and it is defined as follows:

(8) $$PLCC=\frac{\sum_{i}^{N}\left(x_{i}-\bar{x}\right)\left(y_{i}-\bar{y}\right)}{\sqrt{\sum_{i}^{N}{\left(x_{i}-\bar{x}\right)}^{2}}\sqrt{\sum_{i}^{N}{\left(y_{i}-\bar{y}\right)}^{2}}},$$

where *N* is the number of samples, $x_{i}$ and $y_{i}$ are the sample points indexed with *i*, finally $\bar{x}$ and $\bar{y}$ are the means of each sample distribution. Instead, the SROCC estimates the monotonic relationship between the actual and the predicted scores and it is calculated as follows:

(9) $$SROCC=1-\frac{6\sum_{i}^{N}d_{i}^{2}}{N(N^{2}-1)},$$

*N* is the number of samples, and $d_{i}=\left(\mathrm{rank}\left(x_{i}\right)-\mathrm{rank}\left(y_{i}\right)\right)$ is the difference between the two ranks of each sample.

Finally, the RMSE measures score accuracy and it is defined as:

(10) $$RMSE=\sqrt{\frac{1}{N}\sum_{i}^{N}{\left(x_{i}-y_{i}\right)}^{2}},$$

where *N* is again the number of samples, while $x_{i}$ and $y_{i}$ are the sample points indexed with *i*.

For our experiments, we follow the same experimental protocol used in [11,24], which consists of running 100 times the random selection of 60% of training videos, 20% of validation videos, and 20% testing videos. Precisely we exploit the same 100 splits used in [11] that do not prevent the same scene from being both in the training and evaluation sets. Since this fact can cause a bias in resulting performance, for the sake of coherence we train and measure performance of the other methods on the same splits.

## 4. Results

In this section, we report the performance achieved by our method on the four considered databases separately then we compare them with previous NR-VQA methods. Furthermore, we conduct a performance evaluation of the generalization ability of the QSA-VQM in cross-database scenarios, which are more challenging due to different types of contents and degradation characteristics. Finally, an in-depth analysis of the different design choices that have been studied during the designing of the QSA-VQM is reported.

### 4.1. Performance on Single Databases

The experimental results are reported in terms of average PLCC, SROCC and RMSE across the 100 iterations of train-val-test random splits for all the considered databases (CVD2014, KoNViD-1k, LIVE-Qualcomm, and LIVE-VQC). We compare the proposed method with several benchmark methods, namely NIQE [9], BRISQUE [13], V-CORNIA [43], V-BLIINDS [12], HIGRADE [15], TLVQM [24], and VSFA [11]. For the sake of comparison, the same random train-val-test splits were used for all the methods. Table 2 shows the average of the considered metrics and their corresponding standard deviations. We include two results for our QSA-VQM, one representing the final solution “QSA-VQM”, while the other “QSA-VQM (only quality)” illustrates the performance of a variant of the QSA-VQM in which the multi-level feature extractor consists only of the Extractor-Q. We do not estimate the performance for “QSA-VQM (semantics only)”, as this variant of our method would be very similar to the VSFA, which merely extracts semantic features from a model equal to the S-extractor. We, therefore, believe that the results achieved by the VSFA are a good approximation on how the QSA-VQM (semantics only) variant would work, and confirm the effectiveness of the deep semantic features. As it is possible to see the QSA-VQM outperforms the other methods on CVD2014 and KonViD-1k. On the KonViD-1k, the QSA-VQM achieves a higher performance of 0.02 in terms of PLCC and SROCC with respect to the second method, which is still our proposed method considering only quality features, i.e., the QSA-VQM (only quality). On the other hand the QSA-VQM obtains performance equal to the TLVQM on the LIVE-Qualcomm and LIVE-VQC databases almost for all the considered metrics. The fact that these two databases are very challenging is demonstrated by the small mean performance values and the large standard deviation values for all methods. Our method errors are related to: the quality overestimation in videos with camera shake or fast movements; the underestimation of the quality for videos in which the semantic content is not clear or that are taken by night. The higher performance of the TLVQM is then justified by the fact that among the low complexity features, it also considers the motion estimation of key-pixels across frames.

**Figure 5:**
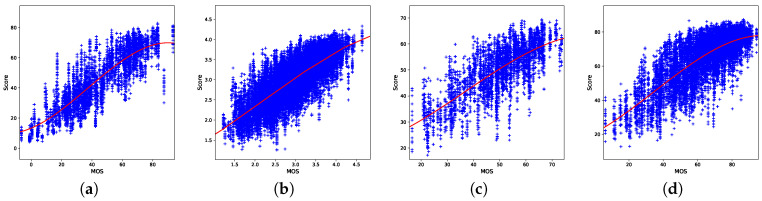
Scatter plots of the predicted scores versus MOS for the four considered databases: (**a**) CVD2014, (**b**) KonViD-1k, (**c**) LIVE-Qualcomm, and (**d**) LIVE-VQC.

Figure 5 shows the scatter plots on the four databases. They report the MOS with respect to the corresponding predicted scores for all the samples considered in the 100 iterations. A logistic regression function is drawn to highlight the silhouette of the fit. We can observe that apart from LIVE-Qualcomm, the other distributions fit well.

**Figure 6:**
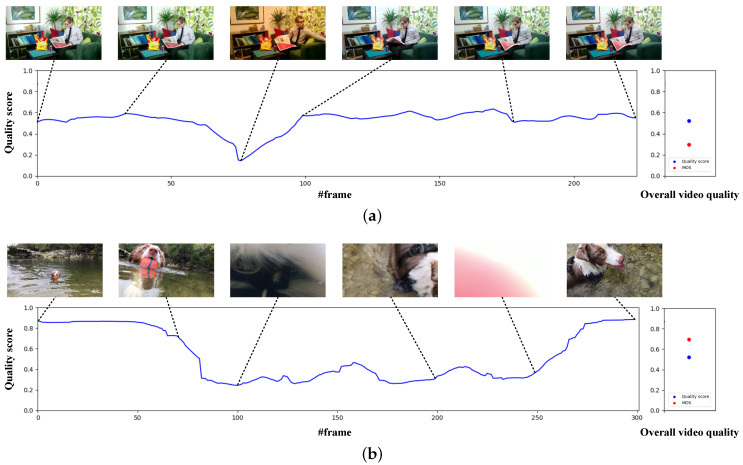
*(Best viewed in colors and magnified.)* Visualization of two samples of video quality overstated or undervalued by the proposed method: (**a**) sample video from CVD2014, and (**b**) video sequence of the LIVE-VQC database. On the left we show the frame-level predictions obtained before the Temporal Average Pooling of the Temporal Hysteresis Pooling layer, while on the right we report the overall video quality MOS and the corresponding predicted score.

In Figure 6, we report two video sequences, belonging to CVD2014 and LIVE-VQC, where our method over- or under- estimate the overall quality. To better understand why the method estimates these quality scores, we provide the predicted quality scores for each frame obtained just before the Temporal Average Pooling and the corresponding video frames. Also, since the ground-truth MOS is provided for the overall video sequence, we compare the latter with the average of the frame-level scores. The quality score for the CVD2014 video sequence has been overestimated by the proposed method. This is probably caused by the fact that there are no motion artifacts, since the video has been acquired using a fixed camera and the subject does not move much, and that the only present artifacts, namely the blocking artifacts, are not very evident. The only impairment that greatly degrades the predicted quality score is a clear yellow color cast around the 60th frame of the sequence. For the LIVE-VQC video, our method predicts a lower quality score than the MOS. In this case, the video shows a very dynamic content due to movements of both the subject and the camera, sometimes the subject is not visible at all and several frames are blurred and overexposed. In our opinion the provided MOS equal to 0.70 does not reflect the objective quality of the video.

### 4.2. Performance Across Databases

The results obtained on different databases separately show the effectiveness of the proposed method. In this set of experiments, we evaluate the robustness and the generalization skills of the QSA-VQM in a cross-database scenario. To this end, for each training database we took the 100 trained models and used them to estimate the quality scores of all the videos from the other databases. Finally we reported the average and the standard deviation on the 100 iterations for each test database. We compare the performance of the proposed method with the two state-of-the-art methods that achieved similar performance on the different databases, namely the TLVQM and the VSFA. Table 3 reports the comparison of QSA-VQM with the other methods when it is trained on a database and tested on the remaining three. We want to highlight that our method generalizes well for all the databases; even for those which did not obtain the state of the art performance in the independent evaluation. It is interesting to notice that the performance of QSA-VQM models trained on LIVE-Qualcomm and tested on the remaining databases are higher even if the training database contains videos affected by only six different in-capture distortions. As already noticed in single database experiments, the performance of the proposed method on videos belonging to LIVE-Qualcomm and LIVE-VQC is low and close to state of the art methods. This is also confirmed in cross-database experiments: the LIVE-VQC is the only database where the QSA-VQM achieves lower performance than both TLVQM and VSFA for all the testing databases.

### 4.3. Computation Time

In this section, we complement the part of performance estimation with that of computational efficiency, which is also crucial for NR-VQA methods. We measure the computational efficiency of several methods and for a fair comparison we conduct all the experiments on the same desktop computer with an Intel Core i7-7700 CPU@3.60GHz, 16 GB DDR4 RAM 2400 MHz, and NVIDIA Titan X Pascal with 3840 CUDA cores. The operating system is Ubuntu 16.04. We compare the computation time of our method with the one of BRISQUE, NIQE, TLVQM, V-CORNIA, V-BLIINDS, and VSFA. Most of the methods are implemented in MATLAB, TLVQM, which has the feature extraction part in MATLAB and the regression part in Python 3.6. VSFA and our method are implemented in Python 3.6 and exploits the PyTorch 1.5.1 framework. For estimating computation time of all methods, we run the original codes using default settings without any modification in CPU. As in [11], we select four test videos with different lengths and different resolutions, namely: 240 frames video with resolution 960×540 pixels, 346 frames at a resolution of 640×480, 467 frames at a resolution of 1280×720, and 450 frames at a resolution of 1920×1080. We repeat the tests ten times and the average computation time (seconds) for each method is shown in Table 4. Our method is faster than VSFA for high resolution videos but it is less efficient than the TLVQM, i.e., the method with the second best performance. At the bottom of the table, we report results in GPU mode for VSFA and QSA-VQM: the only two methods exploiting GPU accelerations among the compared methods. We highlight that these methods in GPU mode can be about 32× faster than the CPU mode.

### 4.4. Ablation Study

In this section, we present the alternative design choices that have been investigated to lead us to the definition of the final model. In particular we compare: the performance of some alternatives to RNN as memory layer; the goodness of the features extracted from a model trained for image quality assessment [44] instead of the estimation of quality attributes as implemented in the QSA-VQM.

Regarding memory layers, the exploration of behavior about different temporal networks on various databases allows estimating the impact of frame-quality-drop in each video. In particular, the effect of using several solutions to model long-term frame dependencies is analyzed, namely Gated Recurrent Unit (GRU) [20], RNN, and a Fully Connected (FC) layer. One of the problems about RNN is about vanishing gradient during the back propagation. In fact, the first network layers could stop learning as the gradient shrinks during back propagation through time. This *short-term memory* phenomena could be significantly reduced using internal mechanisms called gates that regulate the flow of information. GRUs adopt this process to elaborate long sequences of data remembering the most useful past information. So in our experiments we compare the utility of a network that processes long-term dependencies (GRU) between video frames over a network that processes “short-term” dependencies (RNN) and also by linearly combining frame-level features using a simple FC layer. In Table 5, the consequences of using a simple Fully Connected (FC) layer, a GRU, or an RNN in our QSA-VQM are presented. As it is possible to see, the RNN achieves the best performance on almost all the considered databases. On CVD2014, the best result is obtained by the GRU probably because the length of videos is higher than the other considered databases.

**Figure 7:**
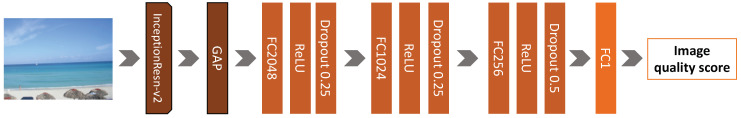
Model trained for image quality assessment to be used as alternative in the Extractor-Q.

About the choice of the extractor for our VQA model, we experiment by changing only the extractorQ with a CNN trained on an image quality assessment dataset, named KonIQ-10k [44], while keeping all the components of the Temporal modeling block. The original model outperforms state of the art in IQA on the KonIQ-10k and the LIVE in the Wild [45] databases. The CNN architecture of the Koncept512 is shown in Figure 7 and consists of an InceptionResNet-v2 [46] head network followed by a Global Average Pooling (GAP) layer, and four Fully Connected (FC) layers, the aim of which is to assign a quality score to each image. We retrain the previous model by using the same hyper-parameters of [44], and the provided dataset split. On the test set images of KonIQ10k, we obtain a PLCC score equal to 0.8797, 0.8588 of SROCC, KROCC equal to 0.6730, and 8.0329 of RMSE after 25 epochs of training. To use the Koncept512 as Extractor-Q of our QSA-VQM, we truncate the architecture to the last convolutional block and then add a GSP layer to obtain the quality features as also explained in Section 2.1. The results obtained on three of the considered databases are presented in Table 6.

## 5. Conclusions

We introduced a novel state of the art NR-VQA method for in the wild videos affected by in-capture distortions, named QSA-VQM. The development process of the final method allows a thorough analysis of the CNNs for the feature extraction process and the most suitable layers for modeling temporal information. In particular, it emerged that the deep features extracted from a CNN pre-trained for characterizing image quality attributes (i.e., sharpness, graininess, lightness, and color saturation) are more effective than features extracted from CNN pre-trained on IQA for frame encoding in video quality assessment. Therefore, the combination of quality features with semantic features has proven effective in producing quality scores as close as possible to human judgment. Furthermore, the use of an RNN layer showed more profitable than the use of GRUs and FC layers for modeling temporal information.

Experiments on four different benchmark databases containing videos with in-capture distortions demonstrate the effectiveness of the proposed method. Finally, the performance evaluation in the cross-database setup has been conducted to point out the robustness and generalization skills of our final method in comparison to other algorithms in the literature.

Based on experimental results, the robustness of the proposed method needs to be improved for better modeling motion characteristics. To this end we will investigate the application of 3D Convolution layers directly on the activation volumes obtained from Extractor-S and Extractor-Q of the Multi-level feature extraction block. Another point that will be explored to improve the performance of the proposed method is the use of a different model for the semantic characterization of video frames, for example, a model that does not model fine-grained content semantics but which instead takes into consideration coarse scene characteristics and the shot type (close-up, medium-, and long- range).

## Figures and Tables

**Table 1:** Overview of the publicly available databases for in-capture video quality assessment. In the column *Device types*: “DSLR” stands for Digital single lens reflex.

Name	No. of Video Sequence	No. of Scenes	No. of Devices	Device Types	Distortion Type	Video Length (s)	Resolution	MOS Range
CVD2014 (2014) [22]	234	5	78	smartphone and DSLR	generic	10–25	640×480 1280×720	−6.50–93.38
KoNViD-1k (2017) [21]	1200	1200	N/A	DSLR	generic	8	960×540	1.22–4.64
LIVE-Qualcomm (2017) [23]	208	54	8	smartphones	specific	15	1920×1080	16.56–73.64
LIVE-VQC (2018) [41]	585	585	101	smartphones	generic	10	404×720 1024×720 1920×1080	0–100

**Table 2:** Mean Pearson’s Linear Correlation Coefficient (PLCC), Spearman’s Rank-order Correlation Coefficient (SROCC), and Root Mean Square Error (RMSE) across 100 train-val-test combinations on the four considered databases. In each column, the best and second-best values are marked in **boldface** and underlined, respectively.

	CVD2014			KonViD-1k		
	PLCC ↑	SROCC ↑	RMSE ↓	PLCC ↑	SROCC ↑	RMSE ↓
NIQE [9]	0.61±0.09	0.58±0.10	17.10±1.5	0.34±0.05	0.34±0.05	0.61±0.03
BRISQUE [13]	0.67±0.09	0.65±0.10	15.90±1.8	0.58±0.04	0.56±0.05	0.52±0.02
V-CORNIA [14]	0.71±0.08	0.68±0.09	15.20±1.6	0.51±0.04	0.51±0.04	0.56±0.02
V-BLIINDS [12]	0.74±0.07	0.73±0.08	14.60±1.6	0.64±0.04	0.65±0.04	0.49±0.02
HIGRADE [15]	0.76±0.06	0.74±0.06	14.20±1.5	0.72±0.03	0.73±0.03	0.44±0.02
TLVQM [24]	0.80±0.04	0.80±0.04	12.89±1.2	0.76±0.02	0.76±0.02	0.42±0.02
VSFA [11]	0.86±0.03	0.86±0.05	11.35±1.4	0.79±0.02	0.78±0.03	0.41±0.03
QSA-VQM (only quality)	0.84±0.05	0.82±0.05	12.15±1.6	0.79±0.02	0.79±0.02	0.40±0.02
QSA-VQM	0.87±0.04	0.86±0.04	11.03±1.4	0.81±0.02	0.81±0.02	0.39±0.03
	**LIVE-Qualcomm**			**LIVE-VQC**		
	**PLCC**↑	**SROCC**↑	**RMSE**↓	**PLCC**↑	**SROCC**↑	**RMSE**↓
NIQE [9]	0.48±0.12	0.46±0.13	10.70±1.3	0.58±0.05	0.56±0.06	13.86±0.7
BRISQUE [13]	0.54±0.10	0.55±0.10	10.30±0.9	0.64±0.06	0.59±0.07	13.10±0.8
V-CORNIA [14]	0.61±0.09	0.56±0.09	9.70±0.9	0.72±0.04	0.67±0.05	11.83±0.7
V-BLIINDS [12]	0.67±0.09	0.60±0.10	9.20±1.0	0.72±0.05	0.69±0.05	11.76±0.8
HIGRADE [15]	0.71±0.08	0.68±0.08	8.60±1.1	0.63±0.06	0.61±0.07	13.03±0.9
TLVQM [24]	0.77±0.06	0.74±0.07	7.62±1.0	0.78±0.04	0.78±0.04	10.75±0.9
VSFA [11]	0.75±0.09	0.71±0.10	8.31±1.1	0.75±0.04	0.69±0.05	11.72±0.9
QSA-VQM (only quality)	0.73±0.08	0.71±0.09	8.46±1.1	0.76±0.04	0.73±0.05	11.41±0.9
QSA-VQM	0.77±0.06	0.74±0.07	7.93±1.0	0.78±0.04	0.74±0.05	11.06±0.8

**Table 3:** SROCC in the Cross-dataset setup. In each column, the best and second-best values are marked in **boldface** and underlined, respectively.

Training	CVD2014			KoNViD-1k		
Testing	LIVE-Qualcomm	KoNViD-1k	LIVE-VQC	CVD2014	LIVE-Qualcomm	LIVE-VQC
TLVQM [24]	0.35±0.08	0.29±0.11	0.41±0.09	0.39±0.08	0.41±0.06	0.50±0.06
VSFA [11]	0.34±0.06	0.55±0.04	0.46±0.04	0.65±0.04	0.60±0.04	0.70±0.01
QSA-VQM (proposed)	0.37±0.06	0.57±0.04	0.41±0.07	0.67±0.04	0.64±0.03	0.66±0.02
**Training**	**LIVE-Qualcomm**			**LIVE-VQC**		
**Testing**	**CVD2014**	**KoNViD-1k**	**LIVE-VQC**	**CVD2014**	**LIVE-Qualcomm**	**KoNViD-1k**
TLVQM [24]	0.48±0.07	0.49±0.04	0.53±0.35	0.49±0.04	0.48±0.04	0.56±0.04
VSFA [11]	0.48±0.07	0.64±0.02	0.63±0.02	0.48±0.06	0.56±0.03	0.67±0.02
QSA-VQM (proposed)	0.53±0.06	0.62±0.02	0.60±0.03	0.47±0.06	0.40±0.07	0.59±0.05

**Table 4:** Computation time comparison in seconds for four videos selected from the considered databases. {xxx}frs@{yyy}p indicates the video frame length and the resolution, respectively.

Mode	Method	240frs@540p	364frs@480p	467frs@720p	450frs@1080p
CPU	BRISQUE [13]	12.69	12.34	41.22	79.81
	NIQE [9]	45.65	41.97	155.90	351.83
	TLVQM [24]	50.73	46.32	136.89	401.44
	V-CORNIA [14]	225.22	325.57	494.24	616.48
	VSFA [11]	269.84	249.21	936.84	2081.84
	V-BLIINDS [12]	382.06	361.39	1391.00	3037.30
	QSA-VQM (proposed)	281.21	265.13	900.72	2012.61
GPU	VSFA [11]	8.85	7.55	27.63	58.48
	QSA-VQM (proposed)	9.70	9.15	25.79	55.27

**Table 5:** Mean PLCC, SROCC, and RMSE across 100 train-val-test combinations on the four considered databases by using different methods for temporal modeling, namely FC, GRU, or RNN.

	CVD2014			KonViD-1k		
	PLCC ↑	SROCC ↑	RMSE ↓	PLCC ↑	SROCC ↑	RMSE ↓
FC	0.8685±0.04	0.8555±0.04	10.9659±1.45	0.8073±0.02	0.8034±0.02	0.3914±0.03
GRU	0.8657±0.04	0.8537±0.04	11.1598±1.61	0.8103±0.02	0.8060±0.02	0.3848±0.02
RNN	0.8658±0.04	0.8545±0.04	11.0406±1.36	0.8109±0.02	0.8069±0.02	0.3884±0.03
	**LIVE-Qualcomm**			**LIVE-VQC**		
	**PLCC**↑	**SROCC**↑	**RMSE**↓	**PLCC**↑	**SROCC**↑	**RMSE**↓
FC	0.7603±0.07	0.7471±0.07	8.1959±0.92	0.7759±0.04	0.7337±0.04	11.2616±0.90
GRU	0.7699±0.07	0.7438±0.07	8.0380±0.94	0.7817±0.04	0.7367±0.05	11.0437±0.84
RNN	0.7736±0.06	0.7439±0.07	7.9321±0.97	0.7822±0.04	0.7369±0.05	11.0591±0.83

**Table 6:** Mean PLCC, SROCC, and RMSE across 100 train-val-test combinations on the four considered databases by replacing the CNN pre-trained for quality attributes with a model trained for image quality assessment.

	PLCC ↑	SROCC ↑	RMSE ↓
CVD2014	0.71±0.07	0.70±0.08	15.40±1.91
KonVid-1k	0.74±0.03	0.74±0.03	0.43±0.02
LIVE-VQC	0.73±0.04	0.69±0.05	11.80±0.67
